# Risk Management in First Aid for Acute Drug Intoxication

**DOI:** 10.3390/ijerph17218021

**Published:** 2020-10-30

**Authors:** Andrea Piccioni, Sara Cicchinelli, Luisa Saviano, Emanuele Gilardi, Christian Zanza, Mattia Brigida, Gianluca Tullo, Gianpietro Volonnino, Marcello Covino, Francesco Franceschi, Raffaele La Russa

**Affiliations:** 1 Emergency Medicine Department, University Polyclinic Foundation A. Gemelli, IRCCS (Scientific Institute for Hospitalization and Treatment), 00168 Rome, Italy; andrea.piccioni@policlinicogemelli.it (A.P.); cicchinelli.sara90@gmail.com (S.C.); luisa.saviano@policlinicogemelli.it (L.S.); mattiabrigida@hotmail.it (M.B.); gianlucatullo@gmail.com (G.T.); marcello.covino@policlinicogemelli.it (M.C.); 2 Emergency-Admission Department, Biomedical Campus, 00128 Rome, Italy; lelegila@outlook.com; 3 Department of Anesthesia, Critical Care and Emergency Medicine, Pietro and Michele Ferrero Hospital, 12051 Verduno, Italy; christian.zanza@live.it; 4 Department of Anatomy, Histology, Forensic Medicine and Orthopedics, Sapienza University, 00161 Rome, Italy; gianpietro.volonnino@uniroma1.it; 5 Emergency Medicine Department, Catholic University of Rome, 00168 Rome, Italy; francesco.franceschi@policlinicogemelli.it

**Keywords:** acute drugs intoxication, emergency department, risk management, medico-legal assessment, temporary observation unit

## Abstract

Drug abuse (cannabis, cocaine, opiates, and synthetic drugs) is an increasing phenomenon, especially in the younger population, thus leading to more cases of intoxication requiring evaluation in the emergency department and subsequent hospitalization. In 2017, 34.2% of students reported having used an illegal psychoactive substance in their lifetime, while 26% reported having done so over the past year. We made a review about the effectiveness of the role of the temporary observation unit in the emergency department to improve management of acute drugs intoxication. We checked medical literature from the last 10 years (2009–2019). The following electronic databases were systematically searched: MEDLINE-PubMed, Web of Science, Scopus, and the Cochrane Central Register of Controlled Trials. Then, a systematic review was carried out according to the Preferred Reporting Items for Systematic Review standards. Intoxicated patients usually display a favorable medical course, few diagnostic and therapeutic interventions, a short stay in the hospital, and, when hospitalization is needed, semi-intensive therapy is a feasible solution; therefore, intoxicated patients are ideal candidates for a temporary observation unit. The emergency department is very important to manage intoxicated patients; however, the hospitalization of these patients is often not necessary.

## 1. Introduction

Acute intoxication is a pathological state of the organism caused by the action of a substance that is toxic by nature or dosage. It is a dynamic process that tends to be short-lived but intense and that can quickly worsen and lead to life-threatening complications. Therefore, it becomes essential to immediately adopt a correct therapeutic approach in the emergency department (ED).

Drug abuse (cannabis, cocaine, opiates, and synthetic drugs) is an increasing phenomenon, especially in the younger population, thus leading to more cases of intoxication requiring evaluation in the ED and subsequent hospitalization.

The 2018 annual report to the Parliament on the state of drug addicts in Italy dedicates some paragraphs to the analysis of the consumption of illegal substances among students. In 2017, 34.2% of students reported having used an illegal psychoactive substance in their lifetime, while 26% reported having done so over the past year. [1]

It is useful to point out that the World Health Organization (WHO) has long recommended, for adolescents up to 16 years, the total abstention from drugs and alcohol [2], taking into account that those who start drinking before the age of 16 have a four times greater risk of developing addiction in adulthood compared to those who start no earlier than 21 years [3,4]. Moreover, these young drinkers are at higher risk to incur other negative consequences, like fatal and non-fatal injuries, blackouts, suicide attempts, unintended pregnancy, sexually transmitted diseases, academic failure, and violence [5].

Often, patients who come to hospital for disorders related to the use of substances are not known to the territorial services and are not followed up for the specific problem [6,7]. In addition to representing an essential tool for the first phase of care, the correct identification of intoxicated patients in ED has wide repercussions in terms of risk management [8,9]. The global evaluation of the patient and his pathology allows to set the best diagnostic-therapeutic path and avoid clinical errors in this category of patients [10,11]. We undertook a narrative review about the effectiveness role of a temporary observation unit (TOU) in ED to improve management of acute drugs intoxication.

### What is a Temporary Observation Unit?

The temporary observation unit (TUO) is the part of the emergency department where patients with acute but non-life-threatening conditions are managed. The typical candidate for this unit is a patient with an expected management time less than 24–48 h and a low workload in terms of nursing, medications use, and diagnostic or operative procedures. After management in the TOU, which can also include specialist consulting (e.g., cardiologist, surgeon, psychiatrist), the patient can be discharged or, if the condition remains unsolved, he can be hospitalized. The patients discharged from the TOU are often addressed to dedicated specialist clinic where they continue their follow-up.

## 2. Materials and Methods

We checked medical literature of the last 10 years from 2009 to 2019 (Table 1). The preferred studies were randomized placebo-control studies, followed by case-control studies, observational (both retrospective or prospective), or systematic reviews.

The following electronic databases were systematically searched: MEDLINE-PubMed, Web of Science, Scopus, and the Cochrane Central Register of Controlled Trials (CENTRAL). The combination of the MeSH terms and the free text words used for the database search were “acute drugs intoxication” and “drugs in emergency department.”

Furthermore, the bibliographies of all the selected articles were checked and all the authors of the included articles were contacted to retrieve unpublished articles or raw data and include as many relevant studies as possible in the analysis.

The present systematic review was carried out according to the Preferred Reporting Items for Systematic Review (PRISMA) standards. Study designs comprised retrospective and prospective studies, letters to the editors, and reviews.

Search Criteria and Critical Appraisal. A systematic literature search and a critical appraisal of the collected studies were conducted. An electronic search of PubMed, Science Direct Scopus, and Excerpta Medica Database (EMBASE) from the inception of these databases to 15 June 2020 was performed.

Methodological appraisal of each study was conducted according to the PRISMA standards, including evaluation of bias. [12] Data collection entailed study selection and data extraction. Five researchers (R.L.R., G.V., G.T., C.Z., and F.F.) independently examined those papers whose title or abstract appeared to be relevant.

Disagreements concerning eligibility between the researchers were resolved by consensus process. No unpublished or grey literature was searched. Data extraction was performed by five investigators (L.S., M.B., E.G., M.C., and C.Z.) and verified by another investigator (A.P.). Only papers in English were included in the search.

**Table 1:** List of studies taken into consideration for this narrative review.

First Author	Type	Year of Publication	No. of Patients	Themes	Findings
Grant BF [3]	Prospective study	1997	27,616	Alcohol use	Starting drinking before the age of 16 years increases the risk of developing alcohol dependence during adulthood.
DeWit Dj [4]	Prospective study	2000	5856	Alcohol use	First use of alcohol at ages 11–14 greatly heightens the risk of progression to the development of alcohol disorders.
Inchley JC [5]	Review	2018	/	Alcohol use	Description of the alcohol-related behavior among European adolescents.
Calle P [6]	Prospective study	2017	487	Alcohol use, illicit substance use	Ethanol remains the most commonly consumed legal substance, but modern illegal recreational substances are also often co-used. The most alarming observation, in particular among MDMA user, was the high-risk poly-drug use.
Manuel JI [7]	Retrospective study	2017	1.2 million	Illicit substance use	Gender-based factors are involved in substance-related ED visits and a better understanding of these differences may help in discharge planning and preventive interventions.
Indig D [8]	Retrospective study	2010	263, 937	Alcohol use, illicit substance use	The study explores the associations between alcohol and drug abuse and polydrug use and their relationship with mental issues.
Margolis A [9]	Prospective study	2016	2019	Illicit substance use	A screening for drug use among patients presenting to the ED of a psychiatric hospital revealed that 9.6% of them used drugs, cannabis was the main substance.
Pomerleau AC [10]	Retrospective study	2012	1207	Illicit substance use	Among 1207 patients presenting in the ED and undergoing a psychiatric evaluation, 14.8% resulted positive to an amphetamine detection test.
Verheij C [11]	Retrospective study	2019	783	Illicit substance use	Intoxications among patients aged 16 years and older are frequently seen at the ED and have substantial healthcare costs.
Di Cesare M [13]	Review	2016	/	Illicit substance use	The annual report to the Italian Ministry of Health identifies differences in drug abuse in patients with mental health issues.
European Drug Report [14]	Review	2019	/	Illicit substance use	Analysis of the latest data on the drug situation and responses to it across the European Union, Norway, and Turkey.
Alho H [15]	Review	2020	/	Illicit substance use	Use of illicit opioids and misuse of prescription opioids are the main causes of drug-related deaths across the world, thus many high-risk opioid users remain outside treatment programs. A wider access to medications for opioid-use disorder (MOUD) may reduce the mortality.
Han Y [16]	Review	2019	/	Illicit substance use	The review explores the recent epidemic and evolution of illicit fentanyl use, its pharmacological mechanisms and side effects, and the potential clinical management and prevention of fentanyl-related overdoses.
Hasegawa K [17]	Retrospective study	2014	731,000	Illicit substance use	The National Hospital Ambulatory Medical Care Survey highlighted that the ED visit rate for opioid overdose quadrupled from 1993 to 2010 in the
Kim HK [18]	Review	2015	/	Illicit substance use	Naloxone is an intrinsically safe drug and may be administered in large doses with minimal clinical effect in non-opioid-dependent patients. However, when administered to opioid-dependent patients, naloxone can result in acute opioid withdrawal. Therefore, it is prudent to use low-dose naloxone (0.04 mg) with appropriate titration to reverse ventilatory depression in opioid-dependent patients.
Chou R [19]	Review	2017	/	Illicit substance use	Low-strength evidence suggested that higher concentration IN naloxone (2 mg/1 mL) is similar in efficacy to IM naloxone (2 mg), with no difference in adverse events.
Pourmand A [20]	Review	2018	/	Illicit substance use	The review discusses the epidemiology, mechanism of action, clinical presentation, and treatment of intoxication for both the common and newest drugs of abuse.
Richards JR [21]	Retrospective study	2017	20,203	Illicit substance use	Comparing the ED accesses of three months of 2016 versus the same three months of 1996, the positivity to methamphetamine screening increased.
Abouchedid R [22]	Prospective study	2017	179	Illicit substance use	Synthetic cannabinoid receptor agonists were found in 10% of this cohort with acute recreational drug toxicity but self-reported in only half of these.
Richards JR [23]	Review	2017	/	Illicit substance use	Cannabinoid hyperemesis syndrome has become more prevalent with increasing cannabis potency and use.
Wong KU [24]	Review	2019	/	Illicit substance use	The review explores the effects of cannabis use in the pediatric population.
Howard K [25]	Review	2016	/	Illicit substance use	The Colorado experience with medical and recreational marijuana legalization has caused an increase of ED accesses. ED physicians must be confident with the marijuana products, their use, and their clinical presentation.
Zhu H [26]	Retrospective study	2016	2,823,321	Illicit substance use	In the period between 2004 and 2011, there has been an increase in cannabis and polydrug abuse, above all in patients aged 12 years or more.
Dines AM [27]	Case series		2198	Illicit substance use	The case series involves 10 European countries, with 2198 cases reported. Among these, 365 regarded the use of cannabis: 76% were males with median age 26 years. Most of the cases regarded as a lone use.
Onyeka IN [28]	Retrospective study	2015	4817	Hospitalization in drug abusers	The leading causes of hospitalization among drug abusers include psychosis, schizophrenia, depression, cardiovascular diseases, hepatitis C, HIV, and other types of hepatitis.
Laine C [29]	Retrospective study	2001	58,243	Hospitalization in drug abusers	HIV-positive drug abusers are more likely to be hospitalized than HIV-negative drug abusers and have longer inpatients days.
Weiss RD [30]	Retrospective study	1992	494	Hospitalization in drug abusers	Among hospitalized drug-abusers, many reported self-medication for depressive symptoms and mood improvement regardless of the drug choice.
Balbinot AD [31]	Retrospective study	2016	76,696	Hospitalization in drug abusers	The introduction of the psychiatric reform has not changed the rate of hospitalizations among drug abusers in Brazil.
Becker D.F [32]	Retrospective study	2007	458	Hospitalization in drug abusers	Development of a predictive model of alcohol and drug abuse in psychiatric hospitalized adolescents.
Gelberg L [33]	Retrospective study	2009	974	Hospitalization in drug abusers	Among homeless women, hospitalization is more likely in drug abusers than in non-drug abusers.
Benson G. [34]	Retrospective study	2019	764	Hospitalization in drug abusers	When hospitalizing a patient with history of alcohol abuse five variables have moderate to strong correlation with the development of severe withdrawal syndrome during the hospitalization.
Maniaci MJ [35]	Retrospective study	2019	251	Alcohol use, illicit substance use	Involuntary hold patients reported increased ED LOS associated with alcohol use, use of barbiturates and screening for urine drug testing. Developing a protocol to help the streamline assessment of alcohol and drug use in this patients’ population may improve the ED LOS.
Piccioni A [36]	Review	2020 (in press)	/	Alcohol use	The acute intoxicated patient is an ideal candidate for temporary unit observation because of a medical course generally shorter than 24 h,
Klein LR [37]	Retrospective study	2017	18,664	Alcohol use	Variables such as diagnostic testing, treatments, and hour of arrival may influence ED LOS in patients with acute ethanol intoxication.
Homma Y [38]	Retrospective study	2018	106	Alcohol use	For the treatment of acute alcohol intoxication (AAI), intravenous crystalloid fluids (IVF) extended ED LOS even after correction for possible confounders. Patients being administered IVF for AAI should be carefully chosen.
Galicia M [39]	Retrospective study	2019	609	Alcohol use, illicit substance use	Co-ingestion of ethanol increases the adverse events of GHB/GBL-intoxicated patients, resulting in greater consciousness impairment, need for care, ICU admission and longer LOS.
Samuels EA [40]	Review	2019	/		The increase in opioid overdose deaths over the past two decades is largely the result of unintentional overdoses. It is not clear that emergency holds would prevent these deaths. The adoption of emergency hold laws as they are currently written is not an evidence-based or justifiable strategy.
Vallersnes OM [41]	Prospective study	2018	1952	Illicit substance use	Nine percent (169/1952) of cases of acute poisoning due to abuse substances, another episode of intoxication re-presented within a week. Patients more likely to re-present were self-discharging patients, homeless patients and those using opioids as toxic agents.
Adam A [42]	Prospective study	2016	631	Alcohol use	Seven years after an admission for alcohol intoxication, patients are likely to present AUDs, substance misuse, mental health disorders, and social problems.
Marshall A [43]	Prospective study	2019	74	Alcohol use, illicit substance use	This study indicates that the awareness and confidence of clinicians can be significantly influenced by targeted training/education, but this must incorporate knowledge translation skills.
Oliver M [44]	Prospective study	2019	173	Alcohol use, illicit substance use	Patients with acute behavioral disorders often have a mental illness history and are commonly intoxicated. These patients have impacts on healthcare resources and pose risks to staff safety, but these patients do not frequently show significant complications.
Lee S [45]	Retrospective study	2008	542	Illicit substance use	Police presentations in the ED are likely to be young males who are unemployed, have past and present alcohol and other drugs use, present after hours. These consumers are likely to have a presenting problem of a psychotic disorder, less likely to have a presenting problem of depression and/or anxiety, and are given a triage code of three or higher.
Brubacher JR [46]	Prospective study	2018	1816	Alcohol use, illicit substance use	Among drivers who are involved in crash accidents alcohol was detected in 15.0%, tetrahydrocannabinol in 7.5%, other recreational drugs in 9.1%, and potentially impairing medications in 20.0%.
La Russa [47]	Retrospective study	2017	/	Adverse drug reaction	This study suggests the need of pharmacogenetic clinical trials in order to better personalize drugs treatment.

## 3. Results

We analyzed 47 studies concerning the use of illicit substances and the access to the emergency department, in particular the abuse of synthetic drugs, cannabis, cocaine and opiates. According to the “Mental Health Report—Data Analysis of the “Mental Health Information System (SISM)” relating to the year 2015 and published in December 2016 by the Italian Ministry of Health, there were 585,000 accesses to the emergency department throughout the country; of these, 39,669 were for “drug addiction” and resulted in hospitalization in 3750 cases (36.9% in psychiatry, 35.6% in general medicine, 3.8% in pediatrics, 1.9% in neurology, 0.6% in general surgery, and 21.2% in other departments) [13].

Summarized data concerning illegal drug use in Europe during 2019 by the European Monitoring Centre for Drugs and Drug Addiction (EMCDDA) can be found in Table 2.

It is estimated that over 96 million adults (aged 15 to 64), or 29%, have tried illicit substances in their lives throughout the European Union. Drug use experiences are more frequently reported by males (57.8 million) than by females (38.3 million). The most used narcotic drug is cannabis (55.4 million males and 36.1 million females), while the estimates are much lower for the lifetime use of cocaine (12.4 million males and 5.7 million females), MDMA (9.3 million males and 4.6 million females) and amphetamines (8.3 million males and 4.1 million females). The claimed levels of cannabis use in life vary considerably between countries, from around 4% of adults in Malta to 45% in France. Among young adults (15–34 years), 19.1 million (16%) have used drugs in the last year, with males being double compared with females (20 vs 11%). Cannabis is the most widely used illicit substance in all age groups. This substance is generally taken by inhaling the smoke; in Europe it is usually mixed with tobacco. Cannabis consumption patterns range from occasional use to regular use through to addiction. An estimated 91.2 million adults in the European Union (15–64 years old), equal to 27.4% of this age group, have tried cannabis throughout their lives. Of these, an estimated 17.5 million young people (15–34 years old), or 14.4% of this age group, have used cannabis over the past year. The prevalence in the last year among young adults ranges from 3.5% in Hungary to 21.8% in France. Among young people who have used cannabis in the past year, the ratio of boys and girls is two to one. If only 15–24 years old are considered, the prevalence of cannabis use is higher: 18% (10.1 million) have used this drug in the last year, and 9.3% (5.2 million) in the last month [14].

## 4. Discussion

### 4.1. Opiates

The amount of seized heroin has increased, the purity of the substance is relatively high and the price relatively low [14]. In Europe, 1.3 million members of the population are at high-risk of opioid abuse and remain outside of dedicated treatment programs. The possible strategies for these patients are reducing rates of prescription opioid use and optimizing opioid prescribing practices; then identify the patients at risk of drug-related deaths; and then a treatment combining opiate receptor full or partial agonist medications for opioid-use disorder (MOUD) like, for example, buprenorphine or methadone at optimal doses, also with psychosocial support, in order to reduce the risk of overdose [15]. Also, highly potent synthetic opioids, notably fentanyl and its derivatives, are also playing an increasing role in both non-fatal and fatal drug overdoses in Europe [16]. Some authors have reported that the ED visits for opioid overdose quadrupled from 1993 to 2010 [17].

Naloxone, an opioid antagonist that has been available for decades, can safely reverse opioid overdose if used promptly and correctly. However, clinicians often overestimate the dose of naloxone needed to achieve the desired clinical outcome, precipitating acute opioid withdrawal syndrome (OWS)**.** Naloxone is an intrinsically safe drug and may be administered in large doses with minimal clinical effect in non-opioid-dependent patients. However, when administered to opioid-dependent patients, naloxone can result in acute opioid withdrawal. Therefore, it is prudent to use low-dose naloxone (0.04 mg) with appropriate titration to reverse the depression of ventilation in this population [18,19].

Data from emergency hospital departments on acute drug-related harm allow us to better understand the influence of drug use on public health in Europe. Hospitalizations for acute drug-related toxicity in selected hospitals in 18 European countries are monitored by the European network of toxicological emergencies (Euro-DEN Plus). The results of this year’s analysis show how the drugs responsible for access to emergency departments can vary in Europe. Stimulants have been associated, for example, with a high number of emergencies, but accesses due to amphetamines have been recorded more frequently in northern and eastern Europe, while cocaine was the prevalent stimulant in southern and western countries [14].

### 4.2. Synthetic Drugs

The 2019 European Drug Report provides an overview of the drug situation in Europe based on the latest available data. The production of synthetic drugs in Europe, although difficult to monitor, seems to be growing, diversifying and becoming more innovative. This expansion is evidenced by recent data documenting an increase in seizures of chemical precursors. The EMCDDA-Europol report on European drug markets, published at the end of 2019, presents an in-depth analysis of these developments. The identification of production laboratories, waste disposal sites, power, and the variety of synthetic drugs available on the European market are fundamental in this sector. The importance of Europe in the world market for synthetic drugs is confirmed by an increasing number of signals, including substantial seizures of substances of various types at the EU borders, the fact that the quantity of MDMA that is seized in Turkey is higher than that seized in the European Union as a whole, and the discovery in Europe of laboratories producing methamphetamine and other synthetic drugs intended for export. The infrastructure for rapidly moving goods from one country to another has been used increasingly for the trafficking of controlled drugs, new psychoactive substances, precursors, and other chemicals essential for the production of drugs in the European Union. The same infrastructure is also sometimes used for the trafficking of synthetic drugs—in particular MDMA, but also other substances—to third countries. The production of synthetic drugs also seems to favor the spread of the use of methamphetamine in new countries of the European Union. Worldwide, methamphetamine represents the biggest challenge in the synthetic drugs sector. In Europe, methamphetamine use was concentrated in some countries where this problem arose long ago. The situation is still largely this, but despite the spread and availability of other stimulants, the analysis of residues in wastewater indicates that this drug is beginning to spread to new countries. The discovery of laboratories also indicates a certain increase in production, as well as production for third-country markets [14].

Popular designer drugs include synthetic cannabinoids (K2, Spice), synthetic cathinones (bath salts), nontraditional opioids (kratom, salvia, Krokodil), opioid analogs (U-47700), and dissociative agents (PCP, ketamine, dextromethorphan) [20].

Since the diffusion of these synthetic drugs is growing, their identification in patients presenting to the ED for acute drug intoxication is also increasing. [21] An observational cohort study enrolling consecutive adult patients presenting to an ED in London (UK) with acute recreational drug toxicity concluded that the synthetic cannabinoid receptor agonists were found in 10% of patients [22].

In the last decade, many physicians did not feel confident in caring for a patient with synthetic drug exposure; today, ED physicians are becoming more prepared since it has become clear that the treatments for most intoxications subsequent to the abuse of synthetic drugs are similar and consist of supportive measures such as ABC stabilization, the maintenance of normal body temperature, control agitation, the management of dehydration and rhabdomyolysis, and guaranteeing the safety of the patient [20].

### 4.3. Cannabis

Cannabis is the most commonly used illicit drug in Europe, and it is generally considered to have low acute toxicity, but there have been reports of acute toxicity associated. The most adverse acute effects are hyperemesis, psychosis, anxiety [23,24]. For these symptoms, patients may seek first aid [25,26].

According to a multi-center case series from 10 different countries published in 2015, it has been observed that most cannabis users were male and often used cannabis with other drugs or alcohol. It was observed that the most acute toxicity was the self-limiting mild neuropsychiatric symptoms and/or vomiting, which do not require treatment and results in only a short length of stay in the ED. There was only one death of cardiac arrest, before admission to the emergency department, also with other cardiovascular fatalities that must be recognized by the emergency physicians [27].

### 4.4. Hospitalization in Drug Abusers 

Illicit drug use is associated with various health problems that often result in hospital admissions. A study by Onyeka et al. has examined a cohort of 4817 drug users and data about their discharge diagnosis. The hospitalization rate was higher among females (84.5%) than males (73.3%), with females having a poorer survival and males having higher h-LOS (hospital length of stay). The leading causes of hospitalization included psychosis, schizophrenia, depression, cardiovascular diseases, hepatitis C, HIV, and other types of hepatitis [28].

The higher trend in hospitalization among HIV-positive drug abusers (55.6% versus 37.5% of HIV-negative) has also been reported by Laine et al.; such patients also show longer inpatients days (27.5 versus 24.5 of HIV-negative) [29].

Drug abuse has been also described as consequence of self-medication for depression. Weiss et al. examined 494 hospitalized drug abusers that reported drug use in response to depressive symptoms and mood improvement regardless of their drug choice [30].

Regarding mental health disorders, a Brazilian study analyzed the effects on the hospitalization of drug abusers after ten years from the psychiatric reform and concluded that there was no reduction in the hospitalization rates or mean occupancy time [31].

Differences in drug abusers’ hospitalization rates are also associated with age, social and ethnic disparities. Some authors have elaborated a prediction model for alcohol and drug abuse in psychiatric hospitalized adolescents belonging to the three most common ethnic groups (European Americans, Latino Americans, and African Americans) in the United States. The model considers seven variables: age, depression, impulsivity, low self-esteem, delinquent predisposition, low peer insecurity, and history of child abuse. The variables have been assessed by using validated scales like the adolescent alcohol involvement scale (AAIS), the drug abuse screening test for adolescents (DAST-A), the Beck depression inventory (BDI), the impulsivity control scale (ICS), the Rosenberg self-esteem scale (RSES), and the Millon adolescent clinical inventory (MACI) [32]. We have not found studies reporting prediction models for addiction in adults, but we can speculate that some risk factors can be important in the adulthood too.

Gelberg et al. have reported that, among homeless women, drug abusers were more likely to be hospitalized than non-drug abusers [33].

After considering the risk factors for hospitalization, the physician should also analyze the consequences of this decision in order to predict and prevent adverse effects. When hospitalizing a patient with history of alcohol abuse, the risk of severe alcohol withdrawal syndrome (SAWS) should be evaluated. A case-control study on 764 patients (382 cases versus 382 controls) has identified five variables with moderate-to-strong association with SAWS risk: fast alcohol screening test, Glasgow modified alcohol withdrawal scale score, AWS at the moment of admission, hours since the last drink, and systolic blood pressure [34]. The identification of patients with high risk soon after their arrival in the ED is useful to establish a prevention strategy with proper therapy.

### 4.5. The Importance of Temporary Observation Unit in the Emergency Room

The intoxicated patient is the ideal candidate to apply the rules of the temporary observation unit (TOU), since his clinical course is often complete within 24 h with a favorable outcome. Moreover, a longer length of stay at the ED is not useful for intoxicated patients [35,36]. For example, a recent retrospective, multi-center, observational study reported that variables such as diagnostic testing, treatments, and hour of arrival may influence and prolong the length of stay at the ED in patients with acute ethanol intoxication [37]. Also, intravenous crystalloid fluid for acute intoxication can prolong the length of stay at the ED [38].

However, potential dangers advise against both imprudent early discharge and admissions to low-intensity wards (psychiatry, general medicine)—and therefore also frequent use of intensive care for the sole surveillance of the patient [39]. For these reasons, it is essential to have some time to decide in which situations to hospitalize or discharge the patient, also considering the implications of potentially involuntary holds [40] and the possibility of early readmissions [41].

The setting (the anamnesis in particular) is made difficult by the panic that the event creates for the patient, family members, and witnesses; all this emotional involvement ends up being transmitted to the healthcare workers in charge of managing the situation. Finally, it should not be forgotten that, in any case, the vast majority of intoxications that require complex treatments are treated in a semi-intensive therapy regime (emergency medicine or emergency area), therefore borne by the emergency doctor.

The identification of acute drug intoxication has important implications in the field of risk management, as it allows us to direct patients toward the best and appropriate therapy [9,40,42,43,44]. Moreover, a correct management of acute substance poisoning is also essential in collaborating with the judicial authority, in cases such as car accidents or violent crimes [45,46]. In fact, a correct care procedure requires the identification of the disease and its causes. The appropriate research of the toxic allows to avoid clinical mistakes such as [47]

- Hospitalization of the patient in the wrong hospital ward;
- Administration of incorrect antidote;
- Administration of drugs having potential synergistic side effects with the molecule taken by the patient.

In a previous study, we have reported that intoxicated patients are at risk of dangerous events but usually display a favorable medical course, few diagnostic and therapeutic interventions, a short stay in the hospital without the need of being hospitalized in a specific ward; and when hospitalization is needed, a semi-intensive therapy is a feasible solution. [36] Therefore, intoxicated patients are ideal candidates for TOU.

However, factors that may prolong the h-LOS, should always be considered. For example, the assumption of a mix of more than one drug or the co-assumption of alcohol and drugs can lead to complications like reduction in consciousness requiring admission to intensive care units. [39] 

## 5. Conclusions

Drug abuse is an increasing phenomenon [13,14,22], also among the young people [5,24,27], with high involvement of the emergency department [17,21,25] and high hospitalization rates [28] and costs. [11] This growing “epidemic” is also spreading because of the misuse of prescribed medications, such as opioids and cannabinoids [15,16].

The length of stay of the patient with acute alcohol intoxication (AAI) in the emergency department (ED-LOS) can be affected by many variables such as the use of diagnostic testing, the need for treatments [38], the hour of arrival [37], and the involuntary holds [35,40].

We present a summary of the most relevant studies published on this topic, starting from epidemiological data, pharmacokinetics and pharmacodynamics, to continue with evidence of potential adverse effects regarding drug abuse. We have analyzed the most important risk factors for hospitalization. However, our review has not identified, to date, a validated protocol that can address the ED physician to choose between discharging and hospitalizing a drug abuser. Nevertheless, many studies of alcohol disorders and drug addiction may be helpful in identifying those patients that, because of their risk factors, may require hospitalization.

Regarding adolescent patients—who make up a large portion of the intoxicated patients—the physician should investigate the age of the starting of alcohol or drug consumption, since the first use of alcohol at a young age (10–14 years) greatly heightens the risk of progression to the development of alcohol disorders. [3,4,5] Moreover, even if ethanol is the most commonly consumed substance, modern illegal recreational substances are also often co-used, and this is why the physician must also investigate about poly-drug use [5,6,8,26]. Poly-drug use, especially with concomitant alcohol consumption, is a “red flag” because it has been reported that this condition is associated with an increase in adverse events, greater consciousness impairment, higher need for care, ICU admission, and longer h-LOS. [39] It is useful, in adolescents, to also evaluate other variables that can predict drug and alcohol abuse, like depression, impulsivity, low self-esteem, delinquent predisposition, low peer insecurity, and history of child abuse [32].

The ED physician has to consider also gender differences [7]. In fact, female drug abusers have higher hospitalization rates and poorer survival, while males have higher h-LO [28]. Moreover, women are more likely to be hospitalized when they are homeless. [33] Young males often come to the ED as police presentations, are often unemployed, and present psychotic disorders [45].

Concomitant diseases that can require hospitalization—like psychosis, schizophrenia, depression, cardiovascular diseases, hepatitis C, HIV, traumatic lesions—have to also be assessed [8,9,10,28,29,30,31,46]. For this reason, the management of the acute intoxicated patient is often multidisciplinary.

Sometimes the ED physician has to postpone the evaluation about risk factors because the patient reaches the emergency department in critical conditions (i.e., consciousness or respiratory impairment, traumatic lesions, psychomotor agitation). It has been demonstrated that awareness and confidence of clinicians can be significantly influenced by targeted training/education. [43] ED physicians should be trained in recognizing the presentation features of the intoxications caused by the main illicit substances and support the vital functions [20,23,25], the indications to the available antidotes [18,19,47], and the prevention of severe withdrawal symptoms [34].

In our experience, for the management of the acute intoxicated patient, we introduce a third option—between discharge and hospitalization—that is part of the emergency department and is managed by the emergency medicine physician: the temporary observation unit (TOU). We have previously reported that the TOU is a good option for the acute alcohol intoxication management [36] since it generally requires less than 24 h with favorable outcome [44]. We suggest that it can be a feasible solution even in the drug abuser, after the exclusion of other medical conditions requiring hospitalization. In the TUO, special attention is given to patients with concomitant mental issues that are evaluated by the consultant psychiatrist, which sets the stage for further clinic follow-up and, if needed, medication therapy. We believe that this strategy may prevent ED low-term and long-term readmission, which have been frequently reported in the literature [41,42]. In fact, many of these patients are not integrated within a support program and have multiple access to the ED [6,7].

From a medico–legal point of view, in order to avoid any medical litigation, hopefully, there should be a standardized protocol in the hospital for the management of intoxicated patients, with adequate training of ED health personnel. In order to reach such standard management, more studies about the prognostic role of the risk factors, should be conducted in the future. In the next years, special effort should be made in educating and training of the ED personnel—possibly including physicians and nurses—hopefully with the development of standardized education programs that include toxicology rudiments. Since the inflow of intoxicated patient is increasing, hospitalization should be reserved to high-risk patients after stabilization in the emergency department. Low-risk intoxicated patients could be managed in a time-effective and resources-effective fashion by the emergency medicine physician in the emergency department, exploiting the potential of the TUO.

## Figures and Tables

**Table 2:** Epidemiological data concerning the use of illicit substances from the European Drug Report 2019 by European Monitoring Centre for Drugs and Drug Addiction (EMCDDA) [14].

ILLICIT SUBSTANCE	EPIDEMIOLOGY (Europe, 2019, Adults 15–64 years old (yo))	EPIDEMIOLOGY (Europe, Lifetime Use, 15–64 yo)	EPIDEMIOLOGY (Europe, 2019, Young Adults 15–34 yo)
Cannabis	24.7 million (7.4%)	91.2 million (27.4%)	17.5 million (14.4%)
Cocaine	3.9 million (1.2%)	18 million (5.4%)	2.6 million (2.1%)
MDMA	2.6 million (0.8%)	13.7 million (4.1%)	2.1 million (1.7%)
Amphetamines	1.7 million (0–5%)	12.4 million (3.7%)	1.2 million (1%)
